# Cesarean Section Among all Deliveries in a Tertiary Care Centre of Nepal: A Descriptive Cross-sectional Study

**DOI:** 10.31729/jnma.6667

**Published:** 2021-09-30

**Authors:** Renuka Tamrakar, Sachin Sapkota, Deekshanta Sitauia, Rohit Thapa, Bandana Pokharel, Suchita Acharya, Aakriti Parajuli

**Affiliations:** 1 Department of Obstetrics and Gynaecology, Chitwan Medicai College, Bharatpur, Nepal

**Keywords:** *cesarean section*, *emergency*, *indications*, *Nepal*

## Abstract

**Introduction:**

Worldwide there is a tremendous increase in cesarean section rate over the last decades which has been a global public health issue. This study aimed to find out the prevalence of cesarean delivery in a tertiary care center of Nepal.

**Methods:**

A descriptive cross-sectional study was conducted among pregnant women at tertiary care centre from 15th September 2019 to 15th October 2020. Ethical clearance was taken from the Institutional Review Committee (Ref: CMC-IRC/077/078-200). Convenience sampling was done to reach the sample size. Basic demographic data, clinical indications and neonatal outcomes were noted. Data entry was done using Statistical Package for the Social Sciences version 20. Point estimate at 95% Confidence Interval was calculated along with frequency and proportion for binary data.

**Results:**

Out of 3193 total deliveries, cesarean deliveries were 1412 (44.22%) at 95% Confidence Interval (42.49-45.94). Among caesarean deliveries 1086 (76.9%) were emergency cesarean sections. Most common indication for cesarean section was fetal distress (24.9%). Among 1437 newborns, 1428 (99.4%) were live births, 1387 (98.2%) were singleton and 801 (55.7%) were male. Nearly one third 418 (29.1%) neonates required neonatal intensive care unit admission and transient tachypnoea of newborns (44.28% in emergency and 60.46% in elective cesarean delivery) was the most common indication for admission.

**Conclusions:**

The prevalence of cesarean delivery was found to be higher than that recommended by the World Health Organisation. Fetal distress was the leading indication for cesarean deliveries.

## INTRODUCTION

Cesarean section (CS) was introduced in late Nineteenth century as a major obstetric intervention to address life-threatening pregnancy and child-birth related complications.^1-2^ It is defined as the birth of foetus through incision in the abdominal wall (laparotomy) and the uterine wall (hysterotomy).^1^

Previous studies from Nepal have reported great variation in rates of CS ranging from 9.5% to 63%.^3,5^ Although the World Health Organization (WHO) has recommended Cesarean Section rate between 10-15% for optimal impact, there has been tremendous increase in its rate worldwide over the last decades.^6^ When medically indicated, CS saves lives of both mother and baby, however, unnecessary C-section may adversely affect maternal, neonatal and infant morbidity and mortality.^2-3^

This study aims to find out the prevalence of Cesarean Delivery in a tertiary care center of Nepal.

## METHODS

A descriptive cross-sectional study was conducted among pregnant women who delivered by Cesarean Section at Chitwan Medical College (CMC) from 15^th^ September 2019 to 15th October 2020. Ethical clearance was taken from the Institutional Review Committee of CMC (Ref: CMC-IRC/077/078-200).

All women who delivered in the centre during the period were included in the study. Informed verbal consent was taken from all the pregnant women. Convenience sampling technique was used and the sample size was calculated using the formula:

n = Z^2^ × p × q / e^2^

  = (1.96)^2^ × 0.344 × 0.656/(0.02 )^2^

  = 2167.28

  = 2168

Where,

n = minimum required sample size
Z = 1.96 at 95% confidence interval
p = prevalence reported by a recent study, 34.4%^7^
q = 1-p
e = margin of error, 2%

Considering non-respondent rate of 10%, total sample size becomes 2385. However, our study included 3193 cases of total deliveries.

We recorded socio-demographic information, obstetric background, significant antenatal events, modes of deliveries and obstetric outcomes in a detailed proforma.

Statistical Package for the Social Sciences (SPSS) version 20 was used for data entry and analysis. Point estimate at 95% Confidence Interval and descriptive statistics were used. Categorical variables were expressed as frequencies whereas continuous variables were expressed as mean±SD or median.

## RESULTS

Out of 3193 pregnant women who delivered at Chitwan Medical College during the study period, 1412 (44.22%) at 95% Confidence Interval (42.49-45.94) women delivered their baby by Cesarean section. The mean age of the women who delivered by CS was 26.44±4.82 years (Range 16-49 years). Among cesarean deliveries, more than half of them were multigravida 752 (53.26%) and most of the deliveries were term deliveries 1228 (87%). Three hundred nineteen (22.6%) women had underlying comorbidities which included Pregnancy Induced Hypertension (PIH) (38.55%), hypothyroidism (15.68%), obstetric cholestasis (13.80%) and Gestational Diabetes Mellitus (GDM) (13.47%). Out of 1412 Cesarean deliveries, more than three-fourths 1086 (76.9%) were emergency Cesarean sections and majority of them 1096 (77.62%) were primary Cesarean deliveries (Table 1).

**Table 1:** Obstetric characteristics by cesarean section (n = 1412).

Gravida	n (%)
Primi	660 (46.74)
Multi	752 (53.26)
**Gestational age**	
Preterm (<37 weeks)	184 (13)
Term (37-42)	1211 (85.8)
Post term (>42 weeks)	17 (1.2)
**Type of delivery**	
Vaginal delivery	1781 (55.78)
Cesarean Section	1412 (44.22)
**Type of CS**	
Emergency CS	1086 (76.9)
Elective CS	326 (23.1)
**Frequency of CS**	
Primary	1096 (77.62)
Repeated	316 (22.38)

Most common indication for CS was found to be fetaldistress 352 (24.9%) followed by previous LSCS 251(17.8%) and meconium-stainedliquor 214 (15.2%)(Table 2)

**Table 2:** Indications for cesarean section in study population (n=1412).

Indications	n (%)
Fetal distress	352 (24.9)
Previous LSCS	251 (17.8)
MSL (Meconium-stained liquor)	214 (15.2)
Failed IOL/Non progression of labor	140 (9.9)
Severe Oligohydramnios	126 (8.9)
Breech	91 (6.4)
PIH	61 (4.3)
Cephalo-Pelvic Disproportion	56 (4.0)
Twins	20 (1.4)
Others	101 (7.2)

Among 1437 newborns, 1428 (99.4%) were live births, 1387 (98.2%) were singleton and 801 (55.7%) were male. Three hundred twenty-three (22.5 %) neonates had low birth weight (LBW). Average weight of newborns delivered by CS was 2902±591gm. Most of them had an Apgar score of six or more both within one minute (1410, 98.1%) and within five minutes (1424, 99.1%) (Table 3).

**Table 3:** Neonatal outcomes after cesarean section. (n=1437)^*^

Birth outcomes	n (%)
Live Birth	1428 (99.4)
Still Birth	9 (0.6)
**Sex**	
Male	801 (55.7)
Female	636 (44.3)
**Fetal Number**	
Singleton	1387 (98.2)
Twins	25 (1.8)
**Weight of Newborn**	
≥2.5 kg	1114 (77.5)
1.5-2.5 kg (LBW)	300 (20.9)
1-1.5 kg (VLBW)^†^	18 (1.3)
<1 kg (ELBW)^‡^	5 (0.3)
**Apgar score at 1 minute**	
0	9 (0.6)
<6	18 (1.3)
≥6	1410 (98.1)
**Apgar score at 5 minutes**	
0	9 (0.6)
<6	4 (0.3)
≥6	1424 (99.1)

* Including 25 twin deliveries

† Very low birth weight

‡ Extremely low birth weight

**Figure 1:**
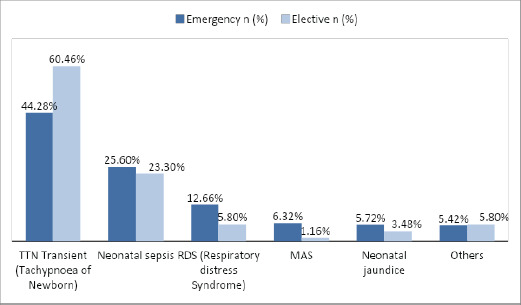
Indications for NICU admission (n = 418).

Nearly one third 418 (29.1%) newborns delivered by CS required NICU admission. Most of them were delivered through emergency CS 331 (79.2%). About one-third 331 (30%) of total babies delivered via emergency CS and a quarter 87 (25.9%) of total babies via elective CS required NICU admission. Most common indications for NICU admission were TTN (44.28% in emergency CS and 60.46% in elective CS) followed by Neonatal Sepsis (25.60% in emergency and 23.30% in elective CS), respiratory distress syndrome (12.66% in emergency CS and 5.8% in elective CS) and Meconium aspiration syndrome (6.32% in emergency CS and 1.16% in elective CS (Figure 1).

## DISCUSSION

The rate of cesarean section has been in rising trend worldwide with most countries exceeding the WHO recommended rate of 10-15%.^6^ This rise in CS has been a matter of debate regarding its indications.

Our study shows the CS rate of 44.22% which is quite towards the higher side as compared to the previous studies carried out in Nepal. Previous studies done in Nepal showed CS rate ranging as high as 63.2% to as low as 9.5%.^4-5^ Recent study conducted in Kathmandu Medical College reported similar rate of CS i.e. 45.81%.^8^ Another study done in Patan academy of health science reported a rise from 38.4% in 2010 to 46.9% in 2014 which is nearly similar to our study.^9^ The study done by K.C. et al. reported that the prevalence of CS delivery has increased by four times from 2001 to 2011.^10^ According to WHO, the incidence of CS in USA, England and China is 32%, 24% and 27% respectively, which is low when compared to our study.^11^ Other Developing countries including India, and regions of South America, also have rates between 25 and 45 %.^12^

In 1985, the WHO stated the rate to be not more than 15% but due to various demographic changes, particularly the increasing maternal age, a target rate of 22% might be more realistic nowadays.^13^ However, above-mentioned studies including ours has shown even higher rate which is a matter of great concern for all obstetricians. The safe motherhood program by the Government of Nepal may be one of the contributing factors for increasing CS in Nepal as it promotes institutional delivery by providing charge-free delivery. This will somehow affect the decision of mothers as well as clinician for CS delivery. Defensive obstetric practice by clinicians and cesarean delivery on maternal request are also a significant factor for the increase in its rate. However, this may have very little influence in tertiary hospitals like ours because very high-risk cases are usually received for which obstetricians are left with no other choice. Our hospital being situated at the center of the city with advanced facilities and equipment is the choice for many other clinicians from periphery to refer the complicated cases.

The CS rate among primigravida was found to be 46.74%. Pradhan et al. also reported a CS rate of 65.9% among primigravidae.^14^ These findings are unacceptably high because of implication of CS on the reproductive career for this group of patients.

The leading indication for CS in our study was fetal distress (24.9%) which was similar to previous studies from Nepal (19.55%, 31.5%).^3,8^ Pradhan et al. reported a high percentage of fetal distress (40.55%) as an indication.^14^ Such result could be due to use of cardiotocography as a main indicator for diagnosing fetal distress. It has been reported that cardiotocography monitoring overestimates fetal distress.^15-16^ Estimation of fetal scalp blood pH is regarded to be gold standard for establishing diagnosis of fetal distress which is not in practice in our institution, not even in other higher tertiary centers over the country. There are range of medical interventions like left lateral positioning, oxygen inhalation to paracervical amnio-infusion for restoration of fetal heart rate.^17^ There is evidence of 70% success rate with paracervical amnio-infusion.^18^ Therefore, caregiver must be encouraged such practice before opting for emergency CS. Overestimation of fetal distress by CTG could be a reason for high CS rate.

Repeat CS (17.8%), second leading cause for CS explored in this study, is a major contributing factor for global excess of CS rates. American college of obstetrician and gynecologists has clearly instructed that previous CS should not be an indication in absence of any other feto-maternal emergencies.^19^ Many studies claim Vaginal Birth After Cesserian Section (VBAC) to be safer alternatives than repeat CS.^12,20^ RCOG recommended that all women previously delivered by one lower segment CS should be offered an opportunity to labor during their next pregnancy by promoting a trial of scar or of labor.^21^Medical literature also suggest that 60-80% of women can safely achieve vaginal delivery.^22^ Rupture of scar was a matter of concern for previous classical cesarean section. However, it became clear that lower segment Cesarean section was not associated with disastrous ruptures.^20^ Regarding our institution, the unwillingness to perform trial of labor after previous CS is probably due to insufficient number of obstetricians as VBAC needs close monitoring, considering CS to be much safer with reduced risk of scar dehiscence or due to maternal preference. Such limited practice of VBAC can be another reason for increased CS rate in our study.

There are few limitations of present study. This is a single-centred study conducted in tertiary level hospitals. So, the findings of this study may not be generalizable. In addition, due to our study design, we were unable to explore the risk factors leading to adverse neonatal outcomes following CS. So, further analytical study is advised to explore those risk factors.

## CONCLUSIONS

The prevalence of cesarean delivery was found to be high in our study. As repeat CS is one of the dominant causes, reduction of primary CS should be given priority. In addition, a comprehensive and evidenced based approach needs to be introduced to monitor the indications of CS and to motivate both provider and recipient for its rational use.
